# Supplementary material for: NXPE1 alters the sialoglycome by acetylating sialic acids in the human colon
Source: Nat Commun. 2025 May 27;16:4912. doi: 10.1038/s41467-025-59671-9 (PMC12216134; doi:10.1038/s41467-025-59671-9)
Supplement: Supplementary file 1 — Supplementary Information [file 41467_2025_59671_MOESM1_ESM.pdf]

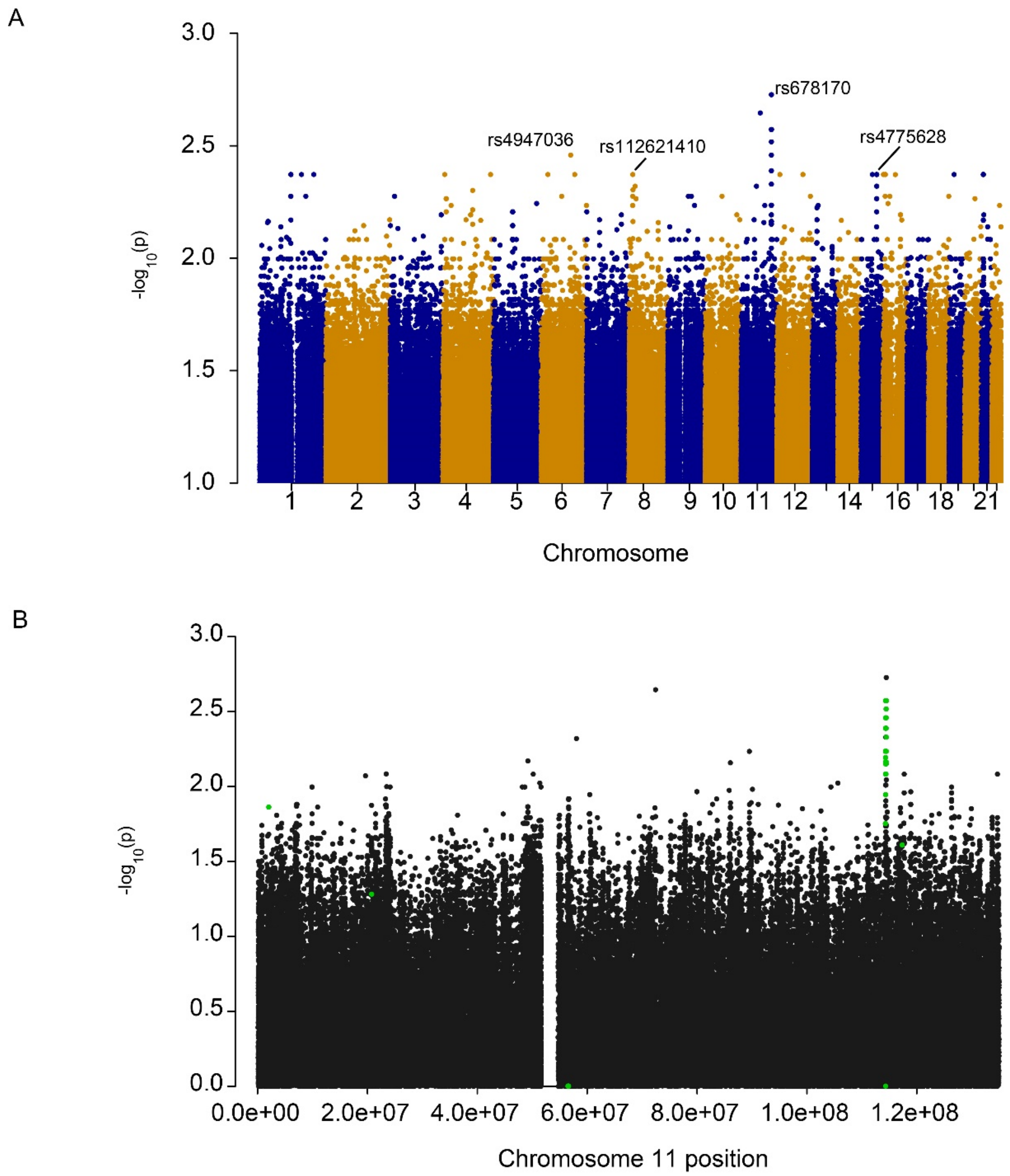

Supplementary Figure 1: A) Manhattan plot showing SNP association with normal colon mPAS staining genome-wide. B) Same as A but showing only chromosome 11. SNPs with perfect genotype-phenotype concordance are highlighted in green, and cluster in a suspected haplotype near 11q23.2.

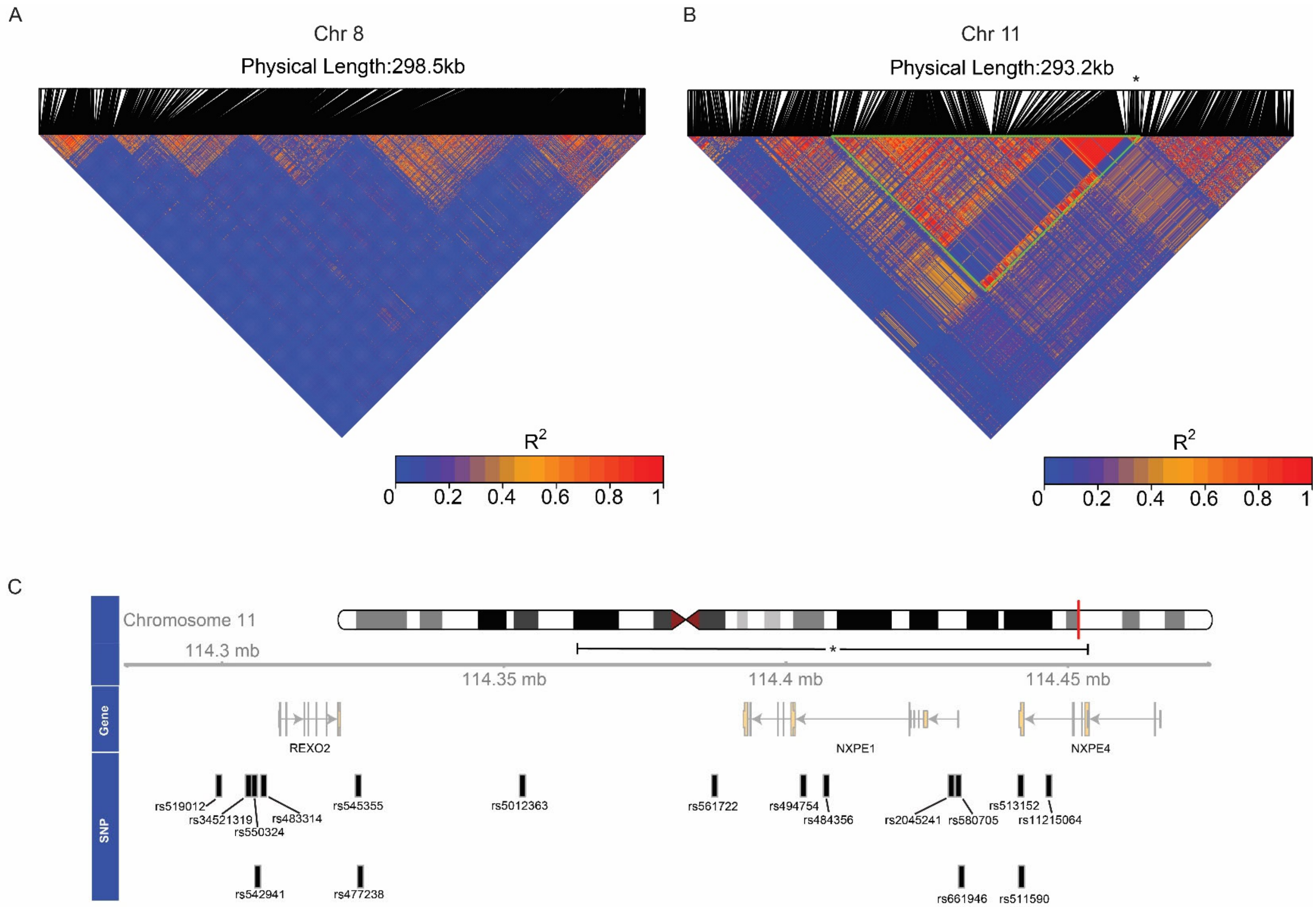

Supplementary Figure 2: A) Linkage disequilibrium plotted as r^2 values for 300kb surrounding the most significant SNP identified in the genome wide association study of mPAS staining near SNP rs112621410 on chromosome 8 and B) rs678170 on chromosome 11. The green highlighted area indicates the region with SNPs demonstrating a perfect concordance between genotype and mPAS phenotype. C) Map showing a zoomed in view of the green highlighted region in B and noting the location of SNPs with perfect concordance between genotype and mPAS staining phenotype. Note that the gene RBM7 is also within the discovered haplotype, but contains no perfectly matching SNPs and thus does not appear in this figure.

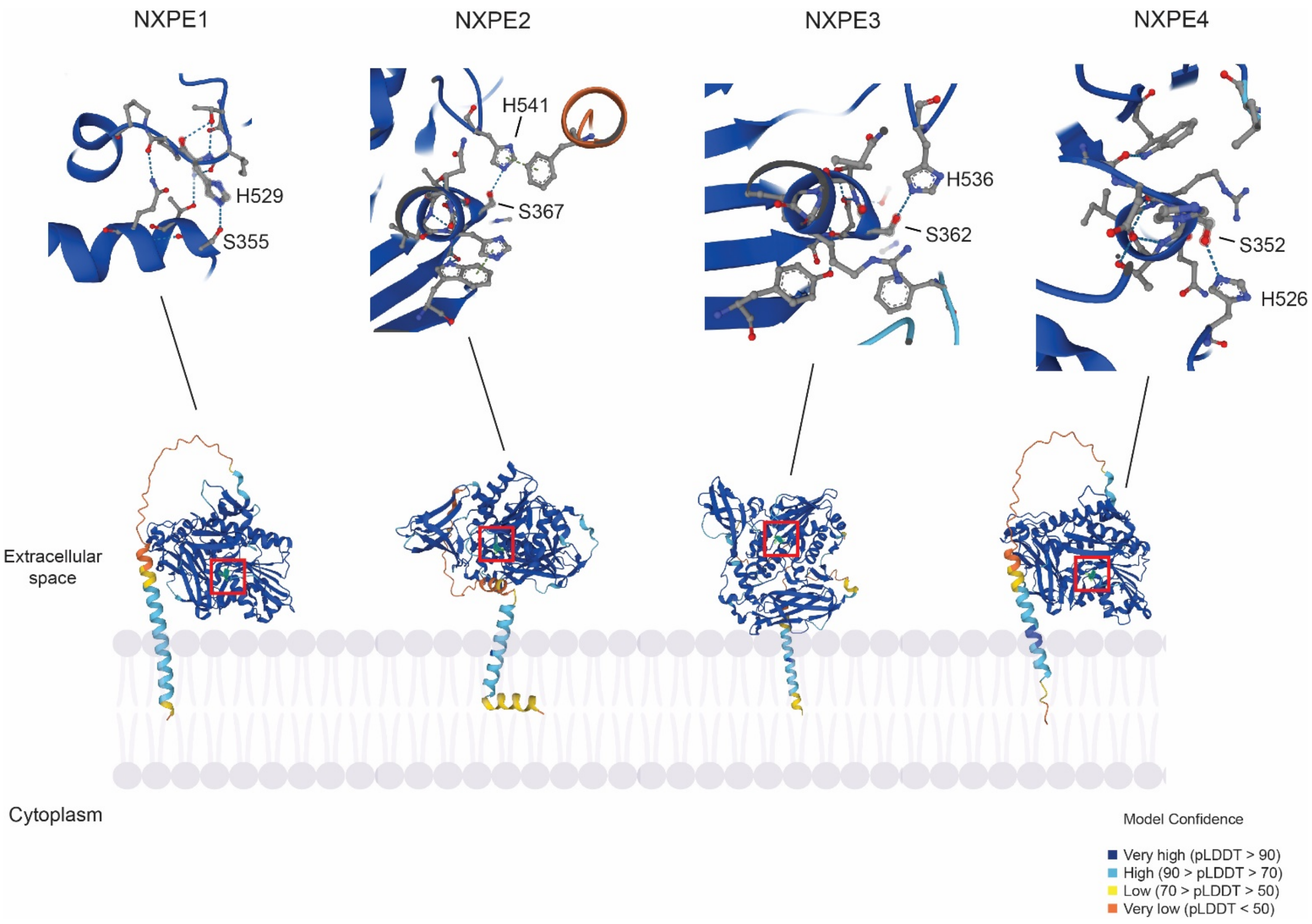

Supplementary Figure 3: *In silico* predicted 3D structures for NXPE family members NXPE1 (Q8N323.2), NXPE2 (Q96DL1.2), NXPE3 (Q969Y0.1) and NXPE4 (Q6UWF7.1). Active site serines and their corresponding histidines are shown (top). All family members are predicted to contain a single transmembrane alpha helix, with the majority of protein in the extracellular (intra-Golgi/ER) space, consistent with glycan location inside and outside the cell. All 4 members contain a conserved Gly-Asp-Ser (GDS) motif, which is also present in known sialic acid acetyltransferase active sites (Figure 2A), with the serine predicted to collocate near a histidine (top). This figure redisplays NXPE1 structural data from Figure 2D. All structures shown in this figure are predictions based on AlphaFold accessed 11/2023.

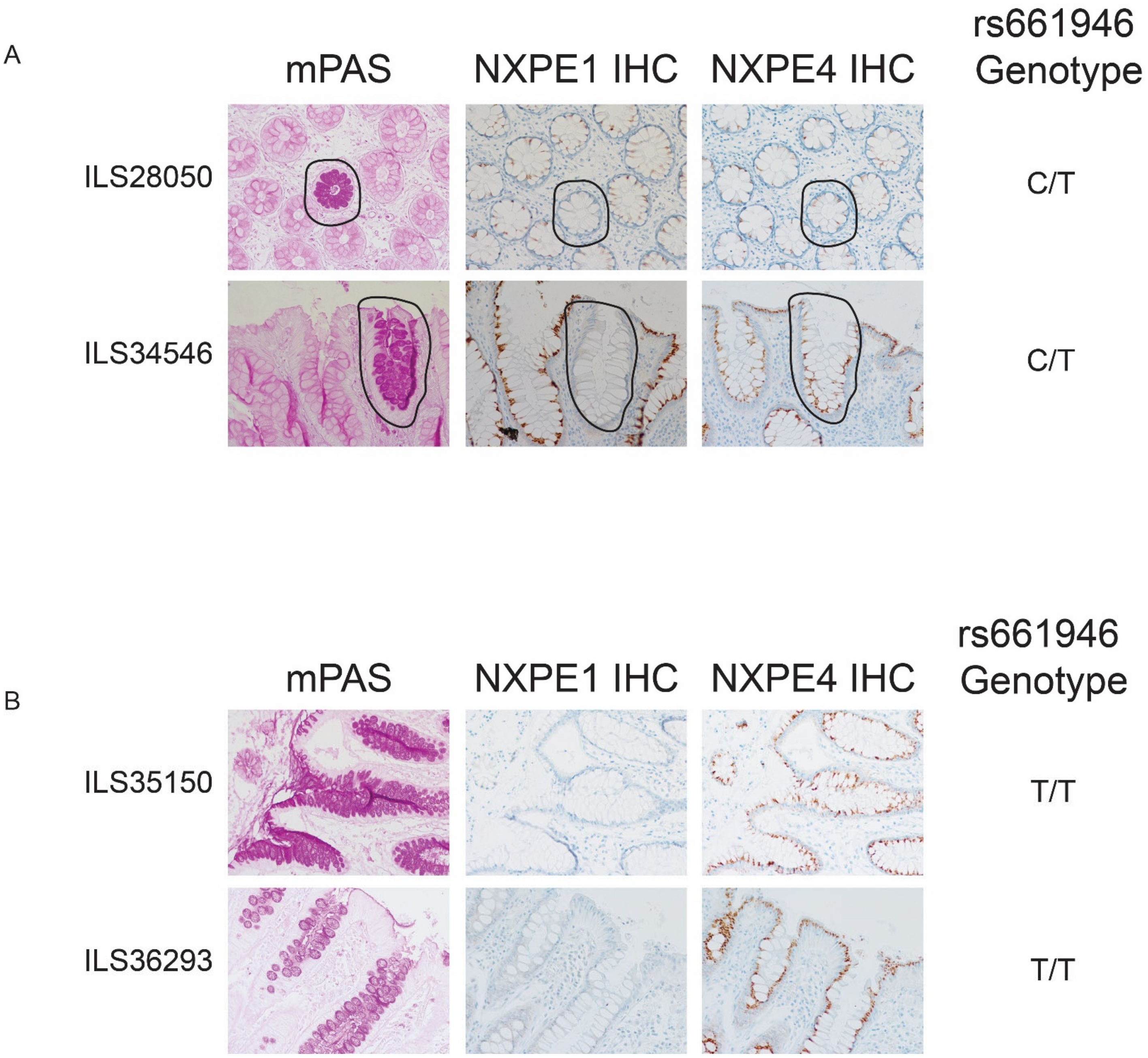

Supplementary Figure 4: A) mPAS, NXPE1 IHC and NXPE4 IHC staining on FFPE normal colon tissue with heterozygous (C/T) and B) homozygous-VAR (T/T) genotypes for SNP rs661946. Heterozygous samples were primarily negative by mPAS, and positive for NXPE1 and NXPE4, but the images chosen show rare cases of spontaneously mPAS positive crypts (circled). Images are shown at 10x, and are representative of stains done on great than 10 different patient samples. This figure provides additional images supporting figure 3A.

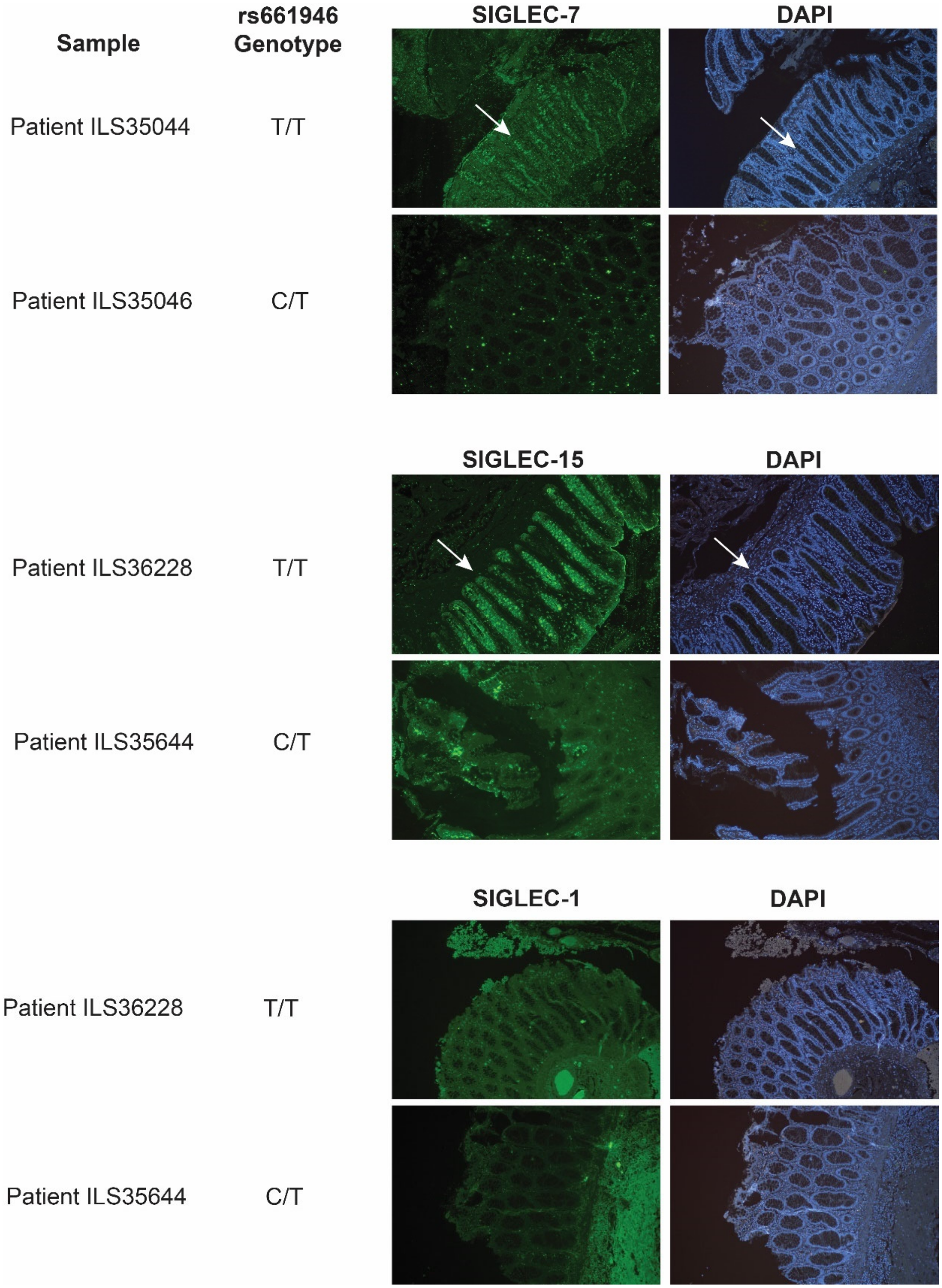

Supplementary Figure 5: SIGLEC-7 (top), SIGLEC-15 (middle) and SIGLEC-1 (bottom) IF staining on normal FFPE colon tissue. The homozygous VAR (T/T) genotype is expected to stain positive for lectin. Arrows show examples of specific staining in goblet cells. SIGLEC-7 displays specific staining of crypt goblet cells, but not as robustly as SIGLEC-15. SIGLEC-1 is shown as a control and does not stain.

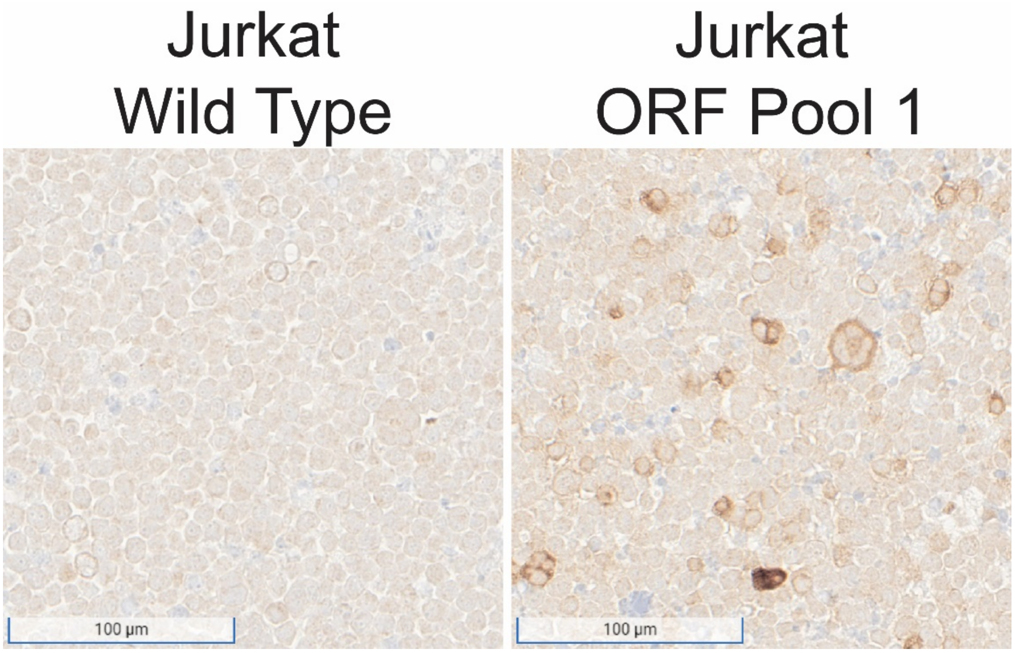

Supplementary Figure 6: IHC of NXPE1 protein on Jurkat parent cells and a pool of Jurkat cells transfected with an NXPE1 open reading frame. Images shown at 10x, and are representative of 2 unique pooled populations of cells that were stained.

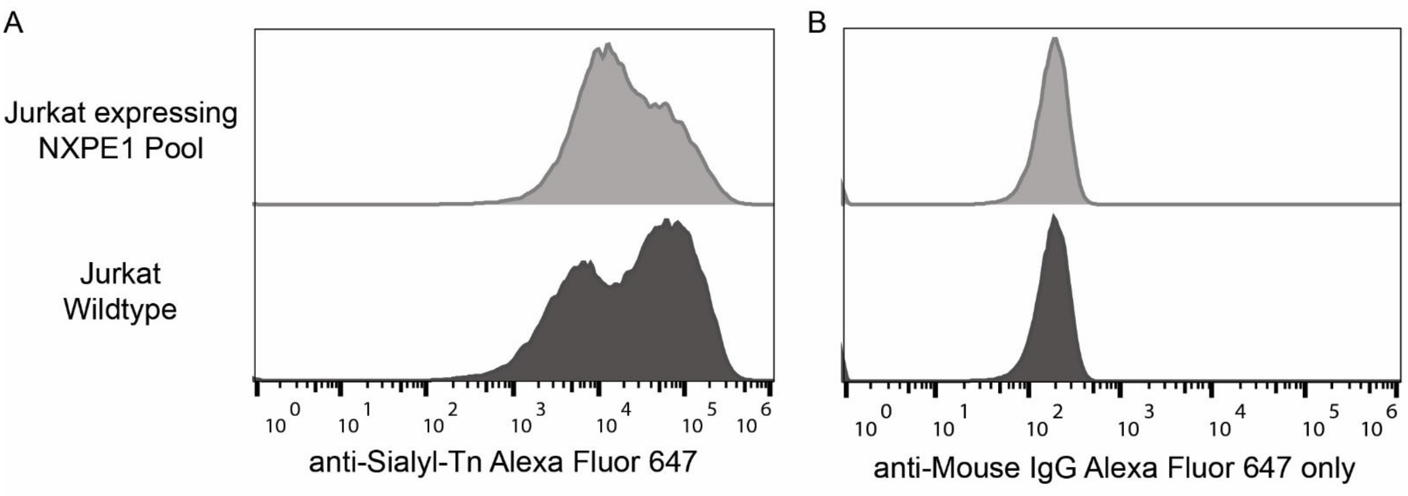

Supplementary Figure 7: Flow cytometry on a pooled population of Jurkat cells infected with lentiviral clones containing a NXPE1 expression cassette using A) an anti-Sialyl-Tn mouse antibody followed by an anti-Mouse IgG Alexa Fluor 647 secondary antibody and B) anti-Mouse IgG Alexa Fluor 647 secondary antibody only. Note that immunostaining and flow cytometry were performed on the same pool of cells as in Figure 4A and 4B.

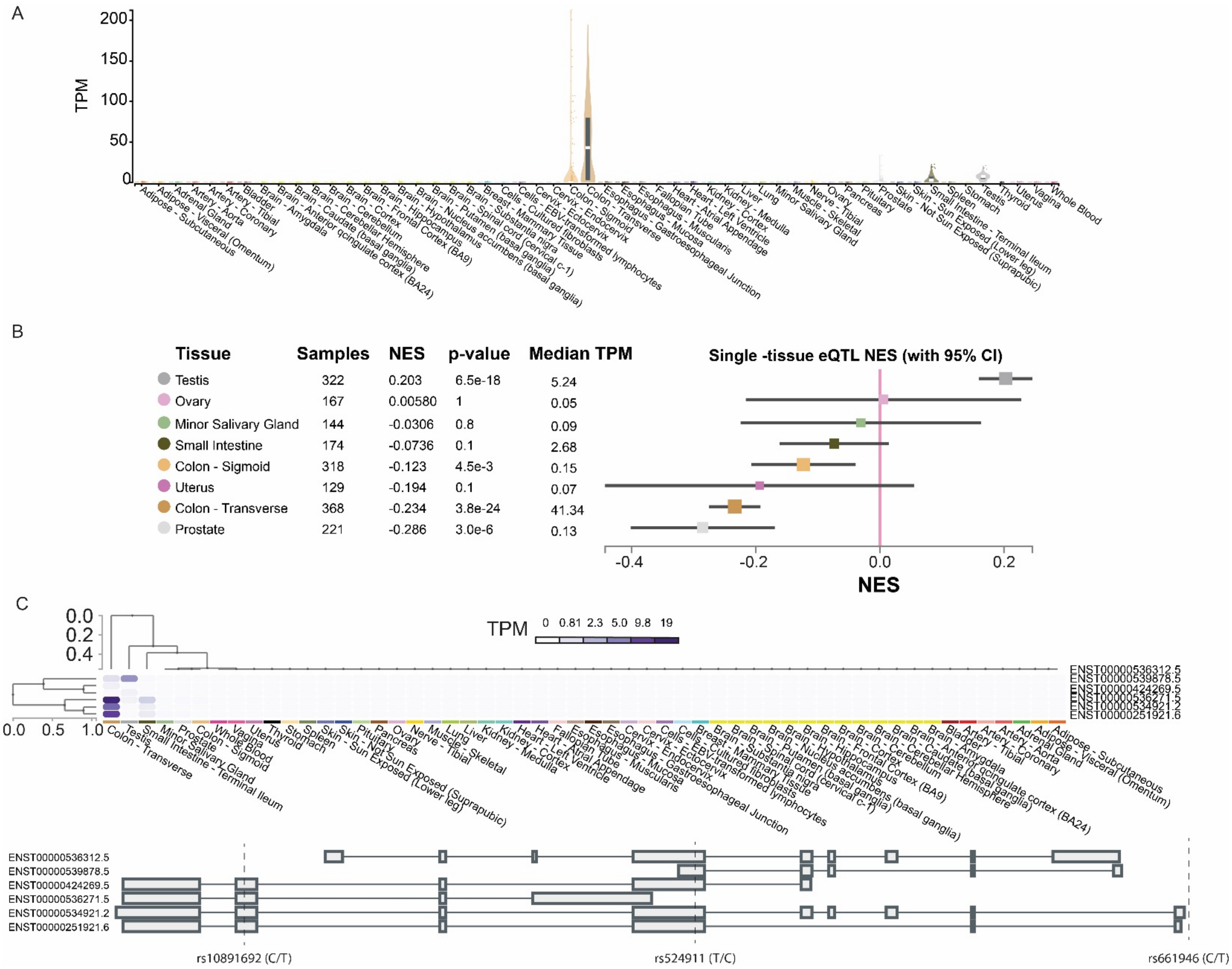

Supplementary Figure 8: A) Violin plots of NXPE1 expression in various tissues. Note the bimodal expression pattern in colon-transverse. B) GTEx eQTL data for *NXPE1* RNA expression and variant rs661946 by tissue type (see acknowledgements). Tissues not shown in this image had no NXPE1 expression. Normalized Effect Size (NES), which is the same as the beta coefficient (regression slope), is calculated by looking at the effect on expression of the variant allele (T/T) relative to the reference allele (C/C), and negative values indicating less expression from the variant (minor) allele. C) NXPE1 isoform expression in various tissues with the two *NXPE1* coding and promoter SNP rs661946 annotated. All data and plots in this supplemental figure were generated via the GTEx portal accessed 3/2025. Refer to GTEx website for information on statistics noted in this figure.

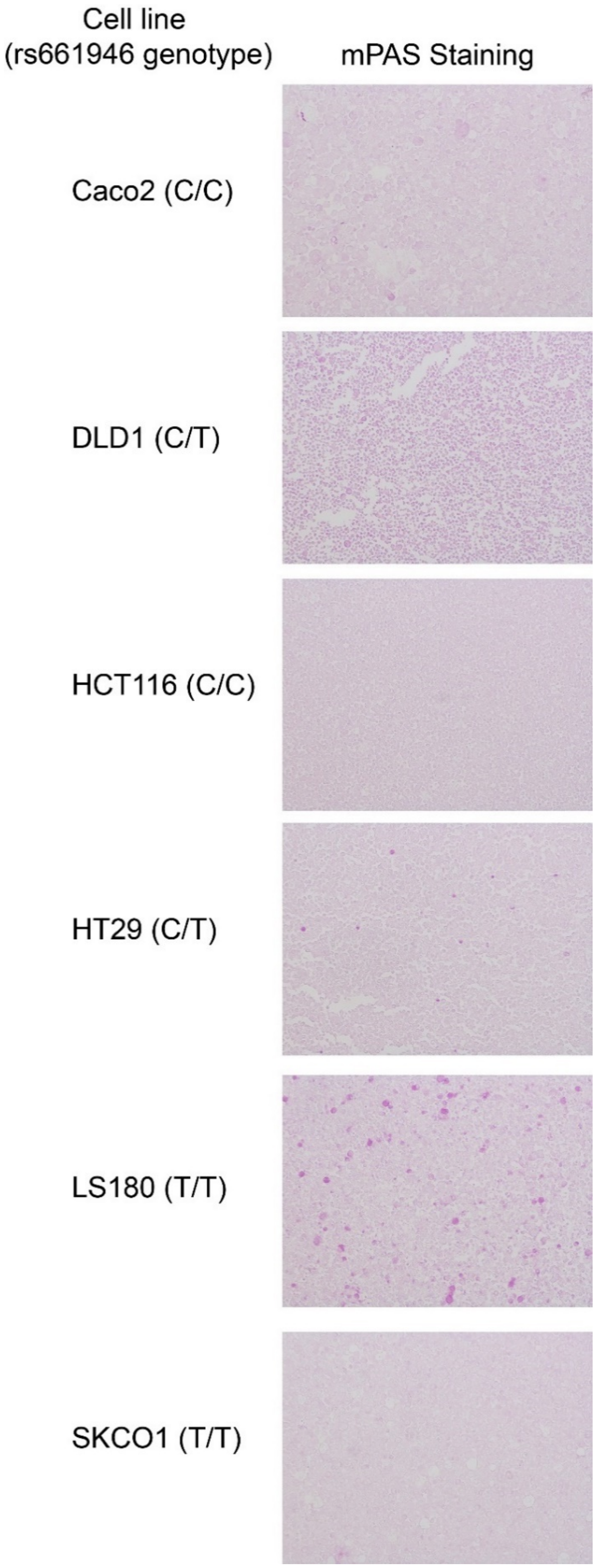

Supplementary Figure 9: mPAS staining on various colon cancer cell lines. SNP rs661946 genotype is noted in parenthesis next to cell line name, T/T genotype is expected to stain positive. Images above shown at 4x and are representative of 3 technical replicates run on each cell line.

LS180
homozygous VAR clone (T/T)

LS180 knock in
heterozygous clone (C/T)

LS180 knock in
homozygous REF clone (C/C)

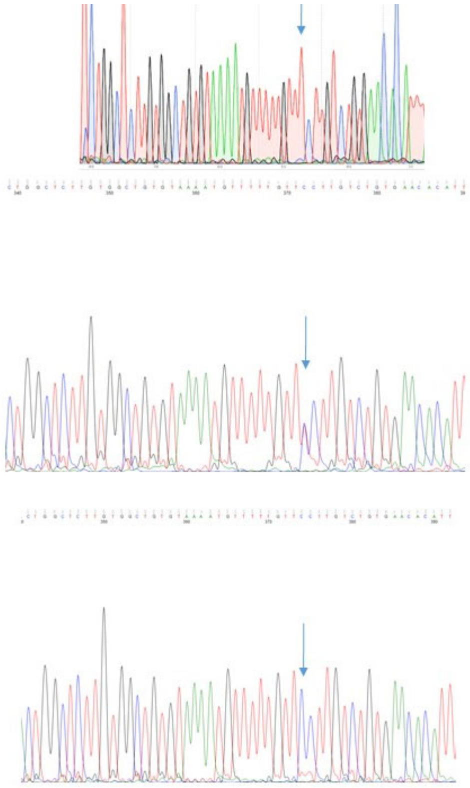

Supplementary Figure 10: Sanger sequencing of *NXPE1* promoter site for LS180 knock in clones where SNP rs661946 (indicated by blue arrow, Hg38 chr11:114,559,887) has been converted to C/T (heterozygous) and C/C (homozygous REF). LS180 parent cell line is T/T (homozygous VAR) and is shown on top. Color indicates base pair identified by sanger sequencing.

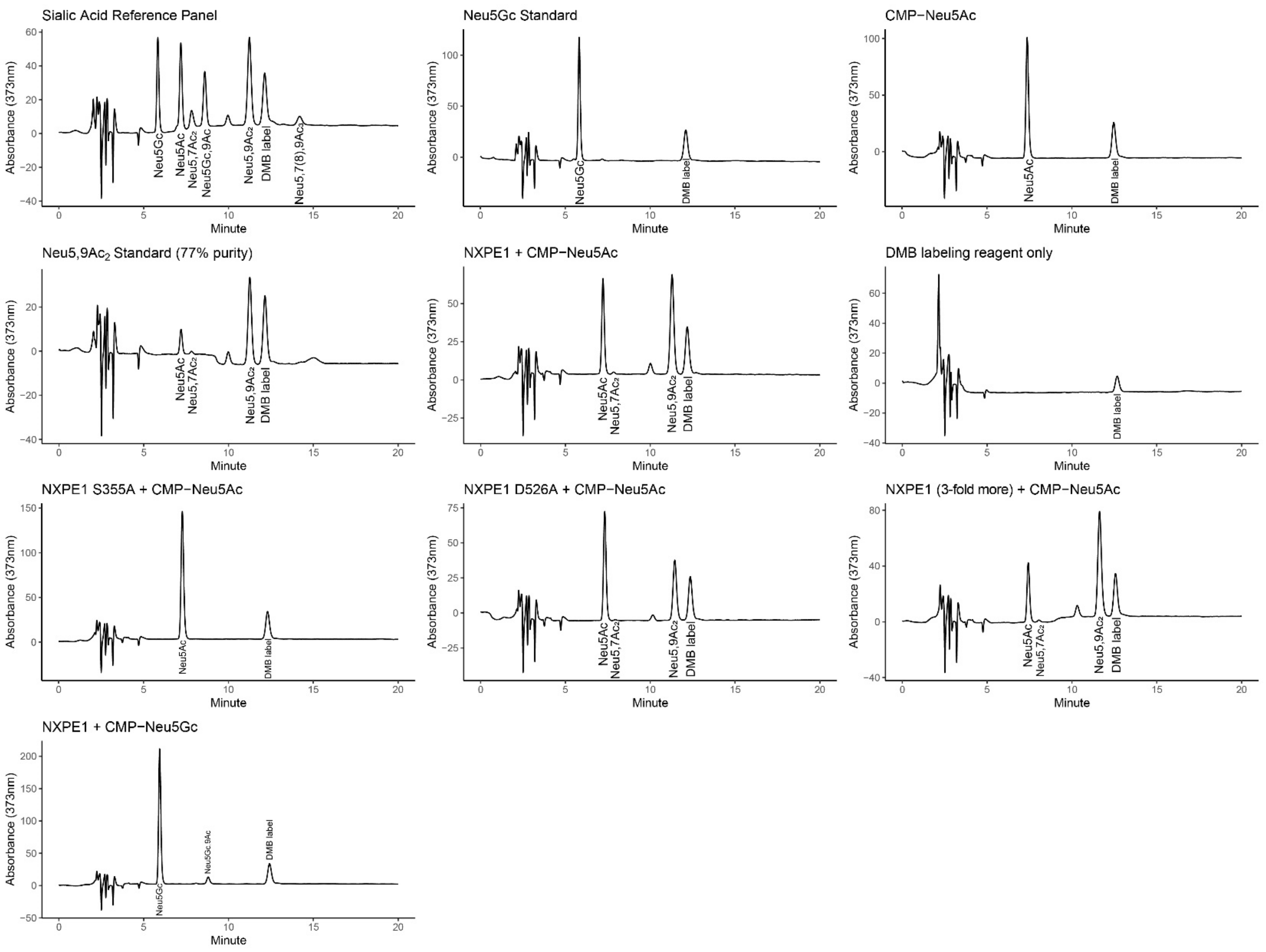

Supplementary Figure 11: Full HPLC spectra showing DMB-based HPLC readout of an enzymatic reaction containing CMP-Neu5Ac and acetyl coenzyme A, with or without the predicted extracellular domain of NXPE1 (AA 60-547). N-glycolylneuraminic acid (Neu5Gc) is a sialic acid that is not synthesized in humans and is shown as a control. Note that some of the images above are also shown in Figure 5A, and that the scale on the y axis changes between spectra.
