# Supplementary material for: NXPE1 alters the sialoglycome by acetylating sialic acids in the human colon
Source: Nat Commun. 2025 May 27;16:4912. doi: 10.1038/s41467-025-59671-9 (PMC12216134; doi:10.1038/s41467-025-59671-9)
Supplement: Supplementary file 2 — Description of Additional Supplementary Files [file 41467_2025_59671_MOESM2_ESM.pdf]

Description of Additional Supplementary Files

Supplementary Data 1: Whole genome sequencing information and file names for fastq files.

Supplementary Data 2: List and information of SNPs identified in WGS as having 2 or less mismatches between the genotype and mPAS phenotype in colorectal samples.

Supplementary Data 3: Colorectal tissue mPAS staining and genotype data for 6 SNPs in our haplotype of interest on chromosome 11 located in coding or promoter regions on validation samples n=91 unique patients. 3x3 tables are shown for statistical testing for SNPs, numbers in red highlight genotype/phenotype mismatches. P values determined by two-sided Fisher's Exact Test and Haldane's exact test used to determine Hardy-Weinberg equilibrium.

Supplementary Data 4: Staining expectations for SNP rs661946 based on genotype for various targets using IHC, IF and mPAS stains.

Supplementary Data 5: GTEx eQTL and sQTL data for genes located in the haplotype identified in our WGS data on chromosome 11. Statistics noted in this table are directly from GTEx, refer to their description for information on how they were obtained.
