# Supplementary material for: NXPE1 alters the sialoglycome by acetylating sialic acids in the human colon
Source: Nat Commun. 2025 May 27;16:4912. doi: 10.1038/s41467-025-59671-9 (PMC12216134; doi:10.1038/s41467-025-59671-9)
Supplement: Supplementary file 8 — Reporting Summary [file 41467_2025_59671_MOESM8_ESM.pdf]

nature portfolio

Corresponding author(s): Kenneth W. Kinzler and Nicolas Wyhs

Last updated by author(s): Apr 7, 2025

Reporting Summary

Nature Portfolio wishes to improve the reproducibility of the work that we publish. This form provides structure for consistency and transparency in reporting. For further information on Nature Portfolio policies, see our Editorial Policies and the Editorial Policy Checklist.

Statistics

For all statistical analyses, confirm that the following items are present in the figure legend, table legend, main text, or Methods section.

n/a	Confirmed	
☐	☒	The exact sample size (*n*) for each experimental group/condition, given as a discrete number and unit of measurement
☐	☒	A statement on whether measurements were taken from distinct samples or whether the same sample was measured repeatedly
☐	☒	The statistical test(s) used AND whether they are one- or two-sided<br>*Only common tests should be described solely by name; describe more complex techniques in the Methods section.*
☒	☐	A description of all covariates tested
☒	☐	A description of any assumptions or corrections, such as tests of normality and adjustment for multiple comparisons
☐	☒	A full description of the statistical parameters including central tendency (e.g. means) or other basic estimates (e.g. regression coefficient) AND variation (e.g. standard deviation) or associated estimates of uncertainty (e.g. confidence intervals)
☐	☒	For null hypothesis testing, the test statistic (e.g. *F*, *t*, *r*) with confidence intervals, effect sizes, degrees of freedom and *P* value noted<br>*Give P values as exact values whenever suitable.*
☒	☐	For Bayesian analysis, information on the choice of priors and Markov chain Monte Carlo settings
☒	☐	For hierarchical and complex designs, identification of the appropriate level for tests and full reporting of outcomes
☒	☐	Estimates of effect sizes (e.g. Cohen's *d*, Pearson's *r*), indicating how they were calculated

Our web collection on statistics for biologists contains articles on many of the points above.

Software and code

Policy information about availability of computer code

Data collection	No software was used
Data analysis	R version 4.3.2 and RStudio (RStudio 2023.09.0+463 "Desert Sunflower" Release (b51c81cc303d4b52b010767e5b30438beb904641, 2023-09-25); PLINK v2.00a6 64-bit and PLINK v1.90b7.2 64-bit (11 Dec 2023) (http://pngu.mgh.harvard.edu/purcell/plink/); AlphaFold; Excel; Whole Genome Analysis - BWA + GATK 2.3.9-Lite; SQL; Variant Effect Scoring Tool (https://www.cravat.us/CRAVAT/); NCBI-BLASTp; COBALT; Jalview

For manuscripts utilizing custom algorithms or software that are central to the research but not yet described in published literature, software must be made available to editors and reviewers. We strongly encourage code deposition in a community repository (e.g. GitHub). See the Nature Portfolio guidelines for submitting code & software for further information.

Data

Policy information about availability of data

All manuscripts must include a data availability statement. This statement should provide the following information, where applicable:

- Accession codes, unique identifiers, or web links for publicly available datasets
- A description of any restrictions on data availability
- For clinical datasets or third party data, please ensure that the statement adheres to our policy

Fastq files for all 21 samples that underwent whole genome sequencing are publicly available at the European Genome-Phenome Archive (https://ega-archive.org/) under accession number EGAS00001007704.

Research involving human participants, their data, or biological material

Policy information about studies with human participants or human data. See also policy information about sex, gender (identity/presentation), and sexual orientation and race, ethnicity and racism.

Reporting on sex and gender	Sex and gender were not considered in the experimental design or review of the data in this manuscript and we expect that the results apply to both male and female humans. The only exception to this statement is sex was considered as a covariate in the genome wide association study and found not to impact the reported genotype-phenotype associations. Sex was not considered during data review as the phenotype studied is observed in both male and female humans at the same frequency based on previous literature. This study only included biological specimen from humans and very generic information about them, such as age, sex and anatomical location the tissue was removed from. Specimen and information about them were provided by the commercial vendor they were purchased from, ILS Bio LLC (Chestertown, MD, USA). Consent regarding data and material from these patients was obtained and managed by ILS Bio LLC.
Reporting on race, ethnicity, or other socially relevant groupings	Socially constructed or relevant variables were not considered in this study. Social groupings were also not made or considered.
Population characteristics	The samples used in this study came from patients with a primarily Asian ethnicity. Most patients had no specific diagnosis as only normal tissue samples were utilized.
Recruitment	The authors did not recruit patients for this study. Samples were obtained commercially.
Ethics oversight	Refer to commercial vendor, ILS Bio LLC.

Note that full information on the approval of the study protocol must also be provided in the manuscript.

Field-specific reporting

Please select the one below that is the best fit for your research. If you are not sure, read the appropriate sections before making your selection.

[x] Life sciences [ ] Behavioural & social sciences [ ] Ecological, evolutionary & environmental sciences

For a reference copy of the document with all sections, see nature.com/documents/nr-reporting-summary-flat.pdf

Life sciences study design

All studies must disclose on these points even when the disclosure is negative.

Sample size	No sample size calculation was performed for this study. Sample sizes were chose based on availability from the sample supplier, and costs/ space related to next generation sequencing.
Data exclusions	Samples without adequate material for both pathology staining and DNA extraction were excluded from the study. This was decided before the study was established.
Replication	The initial GWAS results on 21 samples was confirmed on an independent data set of over 90 samples. All immunological and immunohistochemical staining was confirmed on at least 5 independent samples, representative examples are shown in this manuscript. All flow cytometry and biochemical experiments were confirmed at least twice independently.
Randomization	Samples were prepared, and bar-coded separately and then pooled for next generation sequencing. Validation work (PCR, Sanger sequencing, IHC etc.) was performed on samples placed in plates or slides handled in a random order.
Blinding	Experimenters were not aware of sample phenotype or genotype status during sequencing preparation or data analysis until associations had been completed for the GWAS study. Similarly, experimenters were blinded to genotype/phenotype of samples during validation until results were obtained.

Reporting for specific materials, systems and methods

We require information from authors about some types of materials, experimental systems and methods used in many studies. Here, indicate whether each material, system or method listed is relevant to your study. If you are not sure if a list item applies to your research, read the appropriate section before selecting a response.

Materials & experimental systems

n/a	Involved in the study
☐	☒ Antibodies
☐	☒ Eukaryotic cell lines
☒	☐ Palaeontology and archaeology
☒	☐ Animals and other organisms
☒	☐ Clinical data
☒	☐ Dual use research of concern
☒	☐ Plants

Methods

n/a	Involved in the study
☒	☐ ChIP-seq
☐	☒ Flow cytometry
☒	☐ MRI-based neuroimaging

Antibodies

Antibodies used	Primary antibody, anti-CASD1 (1:100 dilution; Invitrogen, Cat #PA5-60700, Lot #Xi3700359), anti-NXPE4 (1:1000 dilution; Sigma-Aldrich, Cat #HPA042801, Lot #R39941), anti-NXPE1 (1:200 dilution; Santa Cruz Biotechnology, Cat #sc-514349,) or Anti-Sialyl Tn antibody [STn 219] (1:50 dilution for IHC or 2µg/mL for flow staining; Cat #ab115957, Lot 1063860-1, Abcam). recombinant human Siglec-15-Fc (5µg/mL; R&D Systems, USA, Cat #9227-SL-050), Anti-Human IgG Alexa 488 (3µg/mL; Jackson ImmunoResearch, USA, Cat #109-545-170), and Anti-Mouse IgG Alexa 647 (10µg/mL; Cell Signaling, USA, Cat #4410).
Validation	Antibodies were not validated by authors in this study. Specificity for NXPE1, NXPE4, CASD1 antibodies was done by confirming staining primarily in epithelial cells, and for NXPE1 we confirmed IHC results using two unique antibodies.

Eukaryotic cell lines

Policy information about cell lines and Sex and Gender in Research

Cell line source(s)	Jurkat, HEK293T, and LS180 cells were purchased from The American Type Culture Collection (Virginia, USA). Jurkat cells (ATCC, Cat #TIB-152) are male in origin and were grown in RPMI 1640 Medium (ATCC, Cat #30-2001), supplemented with 10% fetal bovine serum (HyClone, Utah, USA, Cat #16777-006) and 1% Penicillin-Streptomycin (Gibco, USA, Cat #15140122). LS180 cells (ATCC, Cat #CL-187) are female in origin and were grown in EMEM (ATCC, Cat #30-2003), supplemented with 10% FBS (HyClone, Utah, USA, Cat #16777-006) and 1% Penicillin-Streptomycin (Gibco, USA, Cat #15140122). HEK-293T cells (ATCC, #CRL-3216) were grown in DMEM (Gibco, USA # 11965092) supplemented with 10% FBS (HyClone, Utah, USA, Cat #16777-006) and 1% Penicillin-Streptomycin (Gibco, USA, Cat #15140122). In vitro cells were maintained at 37°C with 5% CO_2. Mycoplasma testing was performed by The Genetic Resources Core Facility at Johns Hopkins School of Medicine (Maryland, USA).
Authentication	None of the cell lines were authenticated by the authors of this study. All cell lines purchased from ATCC were authenticated by them prior to shipment.
Mycoplasma contamination	None of the cell lines were mycoplasma tested by the authors of this study. All cell lines purchased from ATCC or obtained from the Johns Hopkins Genetic Resources Core Facility were tested prior to shipment or receipt.
Commonly misidentified lines (See ICLAC register)	No commonly misidentified lines were used in this study.

Plants

Seed stocks	Not applicable
Novel plant genotypes	Not applicable
Authentication	Not applicable

Flow Cytometry

Plots

Confirm that:

- [x] The axis labels state the marker and fluorochrome used (e.g. CD4-FITC).
- [x] The axis scales are clearly visible. Include numbers along axes only for bottom left plot of group (a 'group' is an analysis of identical markers).
- [ ] All plots are contour plots with outliers or pseudocolor plots.
- [ ] A numerical value for number of cells or percentage (with statistics) is provided.

Methodology

Sample preparation	Jurkat cells with NXPE1 overexpression were suspended at 1x10^6 cells/mL in staining buffer and incubated with constructs at relevant concentrations for 30 minutes on ice, in the dark. Primary staining was performed with SIGLEC-15 (Acro Biosystems, Cat #SG5-H82E9) monomer at a concentration of 5µg/mL. In our hands, using fresh reagent was preferable for proper staining. Secondary staining was performed with APC-conjugated streptavidin (BioLegend, Cat #405207) at a concentration of 2µg/mL. For anti-Sialyl Tn antibody staining, primary staining was performed with anti-Sialyl Tn antibody (Abcam, Cat #ab115957) at a concentration of 2µg/mL followed by an anti-mouse IgG-Alexa Fluor 647 secondary antibody (Cell Signaling, Cat #4410) at a concentration of 10µg/mL.
Instrument	Attune Nxt Flow Cytometer (Thermofisher)
Software	Flow cytometry data was analyzed using FlowJo v. 10.1 software.
Cell population abundance	*Describe the abundance of the relevant cell populations within post-sort fractions, providing details on the purity of the samples and how it was determined.*
Gating strategy	Single, live cells were isolated using forward and side scatter characteristics. Gating strategy is provided in a separate document attached with this resubmission.

- [x] Tick this box to confirm that a figure exemplifying the gating strategy is provided in the Supplementary Information.
